# An energy landscape approach to understanding variety and robustness in tissue morphogenesis

**DOI:** 10.1007/s10237-019-01222-5

**Published:** 2019-09-07

**Authors:** Hironori Takeda, Yoshitaka Kameo, Yasuhiro Inoue, Taiji Adachi

**Affiliations:** 1 Department of Micro Engineering, Graduate School of Engineering, Kyoto University, 53 Shogoin-Kawahara-cho, Sakyo, Kyoto, 606-8507 Japan; 2 Department of Biosystems Science, Institute for Frontier Life and Medical Sciences, Kyoto University, 53 Shogoin-Kawahara-cho, Sakyo, Kyoto, 606-8507 Japan; 3 Division of Systemic Life Science, Graduate School of Biostudies, Kyoto University, 53 Shogoin-Kawahara-cho, Sakyo, Kyoto, 606-8507 Japan

**Keywords:** Developmental biomechanics, Morphogenesis, Tissue growth, Cell constriction, Energy landscape

## Abstract

During morphogenesis in development, multicellular tissues deform by mechanical forces induced by spatiotemporally regulated cellular activities, such as cell proliferation and constriction. Various morphologies are formed because of various spatiotemporal combinations and sequences of multicellular activities. Despite its potential to variations, morphogenesis is a surprisingly robust process, in which qualitatively similar morphologies are reproducibly formed even under spatiotemporal fluctuation of multicellular activities. To understand these essential characteristics of tissue morphogenesis, which involves the coexistence of various morphologies and robustness of the morphogenetic process, in this study, we propose a novel approach to capture the overall view of morphogenesis from mechanical viewpoints. This approach will enable visualization of the energy landscape, which includes morphogenetic processes induced by admissible histories of cellular activities. This approach was applied to investigate the morphogenesis of a sheet-like tissue with curvature, where it deformed to a concave or convex morphology depending on the history of growth and constriction. Qualitatively different morphologies were produced by bifurcation of the valley in the energy landscape. The depth and steepness of the valley near the stable states represented the degree of robustness to fluctuations of multicellular activities. Furthermore, as a realistic example, we showed an application of this approach to luminal folding observed in the initial stage of intestinal villus formation. This approach will be helpful to understand the mechanism of how various morphologies are formed and how tissues reproducibly achieve specific morphologies.

## Introduction

During morphogenesis in development, mechanical forces resulting from multicellular activities induce tissue deformation (Pearl et al. 2017). Particularly, cell proliferation and constriction are essential activities that generate internal forces. Cell proliferation generates a compressive force in tissues by increasing the cell number and subsequent tissue growth (Ingber 2006; Eiraku et al. 2012). In contrast, cell constriction generates a tensile force under the apical surface of the tissue by activation of actomyosin underneath the cell membrane (Sawyer et al. 2010; Lecuit et al. 2011; Heisenberg and Bellaiche 2013).

In morphogenesis, a variety of morphologies such as sheet, shell, and tube are formed because of various spatiotemporal combinations and sequences of multicellular activities (Sawyer et al. 2010; Filas et al. 2012a). For example, sheet-like tissues deform into qualitatively different shapes depending on the spatial pattern of contractile cells as observed in experimental studies (Filas et al. 2012a; Kondo and Hayashi 2015) and supported by mathematical studies (Filas et al. 2012a; Misra et al. 2016; Inoue et al. 2017). Despite its potential of variation, morphogenesis is a surprisingly robust process, in which qualitatively similar morphologies are reproducibly formed even under spatiotemporal fluctuations caused by multicellular activities (Goodwin et al. 1993; Conte et al. 2009; Davidson 2017). For example, even when the constriction driving tissue deformation is fluctuated at a particular stage, morphogenesis proceeds normally (Royou et al. 2004; Eiraku et al. 2011; Krajcovic and Minden 2012). Different spatiotemporal activities induce different morphologies, but these morphogenetic processes remain robust in spite of their fluctuations. To understand this coexistence of contradictory characteristics, the variety and robustness in morphogenesis, development of a universal method to capture these features on the same basis is required. Since the process of multicellular tissue deformation in morphogenesis is induced by mechanical effects of multicellular activities, an energy-based approach from a mechanical viewpoint would be suitable.

To understand how morphogenesis possesses the characteristics of both variety and robustness, in this study, we propose a novel approach to capture an overall view of morphogenesis by describing an energy landscape. We targeted morphogenesis of a sheet-like tissue caused by proliferation and constriction generating internal compressive and tensile stresses in the tissue and analyzed the total strain energy in the whole tissue by performing finite element analysis (FEA). In the undulating energy landscape, the emergence of various morphologies was represented as a bifurcation into multiple stable states. The robustness of morphogenesis to fluctuation of multicellular activities was represented as the stability of these states. By following the path that represents temporal changes in the mechanical state in the landscape, we demonstrated and explained how qualitatively different morphologies emerged depending on the history of the multicellular activities, using a sheet-like tissue with curvature by showing the commonly observed bifurcation of invagination or evagination due to cell constriction and tissue growth. Furthermore, as a realistic example, we showed an application of this approach to luminal folding observed in the initial stage of intestinal villus formation. This approach can provide a universal understanding of the variety of tissue morphologies and the robustness of morphogenesis.

## Continuum mechanics models

To investigate how growth and constriction mechanically affect deformation of a sheet-like tissue, we performed finite element analysis based on the nonlinear continuum mechanics, considering a large deformation and nonlinear material properties of biological tissue. Tissue-scale volumetric growth caused by cellular proliferation is characterized through a multiplicative decomposition of deformation gradient ${\mathbf{F}}$:

1 $${\mathbf{F}} = {\mathbf{F}}^{\text{e}} {\mathbf{F}}^{\text{g}}$$

where ${\mathbf{F}}^{\text{e}}$ and ${\mathbf{F}}^{\text{g}}$ denote an elastic part and a growth part, respectively (Rodriguez et al. 1994; Menzel and Kuhl 2012). To analyze an idealized sheet-like tissue model, we postulated a planar isotropic growth, because epithelial cells proliferate while maintaining a single-layer structure. The growth tensor ${\mathbf{F}}^{\text{g}}$ is described by

2 $${\mathbf{F}}^{\text{g}} = \theta {\mathbf{I}} + \left( {1 - \theta } \right)\varvec{n} \otimes \varvec{n},$$

where the directional unit vector ***n*** is defined as the direction of out-of-plane growth and $\theta ( > \,1)$ denotes in-plane growth stretch perpendicular to ***n***. To analyze tubular folding in the initial stage of intestinal villus formation, we assumed circumferential growth. The growth tensor ${\mathbf{F}}^{\text{g}}$ is described by

3 $${\mathbf{F}}^{\text{g}} = {\mathbf{I}} + \left( {\theta - 1} \right)\varvec{\zeta}\otimes\varvec{\zeta},$$

where the directional unit vector $\varvec{\zeta}$ is defined as the circumferential direction and $\theta ( > 1)$ denotes growth stretch parallel to $\varvec{\zeta}$.

To model directional constriction of epithelial cells, we adopted the active stress model (Kida and Adachi 2015). In this model, the total elastic energy density $\psi^{\text{e}}$ is assumed to be the sum of passive free energy density $\psi_{\text{pas}}^{\text{e}}$ and active free energy density $\psi_{\text{act}}^{\text{e}}$ as follows:

4 $$\psi^{\text{e}} = \psi_{\text{pas}}^{\text{e}} + \psi_{\text{act}}^{\text{e}}$$

We modeled the material property of epithelial tissue as a hyperelastic material. We chose a Neo-Hookean model as the passive free energy

5 $$\psi_{\text{pas}}^{\text{e}} = \frac{\lambda }{8}\ln^{2} I_{3} + \frac{\mu }{2}\left( {I_{1} - 3 - \ln I_{3} } \right)$$

where $I_{1} = {\text{tr}}{\mathbf{C}}^{\text{e}}$ and $I_{3} = { \det }{\mathbf{C}}^{\text{e}}$ are invariants of an elastic Cauchy–Green tensor ${\mathbf{C}}^{\text{e}} = {\mathbf{F}}^{\text{eT}} {\mathbf{F}}^{\text{e}}$. $\lambda$ and $\mu$ are Lame’s constants. The strain energy $\varPsi_{\text{pas}}^{\text{e}}$ is calculated using a volume integral of $\psi_{\text{pas}}^{\text{e}}$ as follows:

6 $$\varPsi_{\text{pas}}^{\text{e}} = \int {\psi_{\text{pas}}^{\text{e}} {\text{dV}}}$$

To represent planar isotropic constriction, we defined two constriction directions that were perpendicular to each other. The directional unit vector $\varvec{m}_{0,i}$ (*i* = 1, 2) in the reference configuration and $\varvec{m}_{i}$ in the current configuration are defined as the directions in which a tissue constricts. The relation between $\varvec{m}_{0,i}$ and $\varvec{m}_{i}$ is described by

7 $${\mathbf{F}}^{\text{e}} \varvec{m}_{0,i} = \lambda_{{{\text{m}},i}} \varvec{m}_{i}$$

where $\lambda_{{{\text{m}},i}}$ is the stretch of $\varvec{m}_{0,i}$. The free energy function associated with active stress is described by

8 $$\psi_{\text{act}}^{\text{e}} = \mathop \sum \limits_{i = 1}^{2} A\left\{ {\lambda_{m,i} + \frac{1}{3}\frac{{\left( {\lambda_{\hbox{max} } - \lambda_{m,i} } \right)^{3} }}{{\left( {\lambda_{\hbox{max} } - \lambda_{0} } \right)^{2} }}} \right\}$$

where *A* is a degree of magnitude of active stress ($A \ge 0$), and $\lambda_{ \hbox{max} }$ and $\lambda_{0}$ denote the stretch at which maximum and minimum active stress are generated, respectively. The constitutive relationship between active stress and stretch is shown in “Appendix” (Fig. 5).

## Results

### Idealized model for tissue morphogenesis of a curved sheet

#### Simulation conditions

To elucidate the concept of an energy landscape approach, we investigated variety and robustness of morphogenesis using an idealized model of curved sheet-like tissue. Despite the idealized model, we believe that this model realistically captures essential behaviors of sheet-like tissue morphogenesis with cell proliferation and constriction, such as during furrow formation in *Drosophila* (Royou et al. 2004; Krajcovic and Minden 2012), neurulation in *Chick* and *Xenopus* (Nishimura et al. 2012; Butler and Wallingford 2018), and optic-cup formation in *Zebrafish* (Nicolas-Perez et al. 2016; Martinez-Morales et al. 2017).

**Fig. 1:**
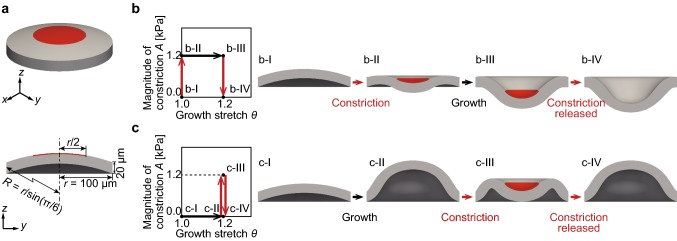
Simulations of tissue morphogenesis caused by growth and constriction, showing its dependency on history of multicellular activities. **a** Top view and sectional view of the initial geometry of the sheet-like tissue. Volumetric growth occurs planar isotropically in the whole tissue. Constriction occurs planer isotropically in the red region. **b**, **c** Cross-sectional view of morphological changes when constriction occurred in the red region before growth (**b**) and after growth (**c**). The histories of change in the growth stretch *θ* and magnitude of constriction *A* [kPa] are shown on the left

We constructed a finite element (FE) model of an idealized circular sheet-like tissue that had an initial curvature (Fig. 1a). We set the distance from the central axis to the boundary surface as *r* = 100 μm with a curvature radius $R = r/\sin (\pi /6)$. Tissue thickness was 20 μm. We chose the elastic parameters $\lambda = 621\,{\text{Pa}}$ and $\mu = 69\,{\text{Pa}}$ corresponding to Young’s modulus $E = 200\,{\text{Pa}}$ and Poisson’s ratio $\nu = 0.45$, respectively.

We analyzed tissue deformation by performing FEA based on continuum mechanical models. Cell proliferation was modeled as the volumetric growth based on a growth model (Menzel and Kuhl 2012; Rodriguez et al. 1994). The planar isotropic growth occurs in the whole tissue. The out-of-plane growth direction $\varvec{n}$ in Eq. (2) was set to be perpendicular to the surface of the sheet-like tissue. An increase in the growth stretch $\theta \left( { \ge \,1} \right)$ in Eq. (2) represents planar isotropic growth in the whole tissue. Cell constriction was modeled as the energy function of generating contractile forces based on an active stress model (Kida and Adachi 2015). An increase in the magnitude of constriction $A \left( { \ge \,0\;{\text{kPa}}} \right)$ represents planar isotropic constriction. The constriction occurs within the center circle on the tissue surface, whose radius is $r/2$ (Fig. 1a). The boundary surface at radius *r* = 100 μm was fixed, that is, all degrees of freedom of displacement were fixed. The parameters in Eq. (7) are $\lambda_{ \hbox{max} } = 1.5$ and $\lambda_{0} = 0.2$. The strains obtained in the present FEA simulation ranged from about 0.6–1.0. In this range, the active second Piola–Kirchhoff stress was less sensitive to the value of stretch than the magnitude of constriction *A*; the active stress is mainly governed by the magnitude of constriction *A*. Thus, the constitutive behavior of active stress does not significantly affect the qualitative profile of the energy landscape.

#### Tissue morphologies depending on the history of growth and constriction

We investigated typical cases in which growth and constriction occurred sequentially. We considered two cases—constriction occurs before growth, and constriction occurs after growth—both of which were followed by the release of constriction to examine tissue morphologies in a constriction-free configuration.

**Fig. 2:**
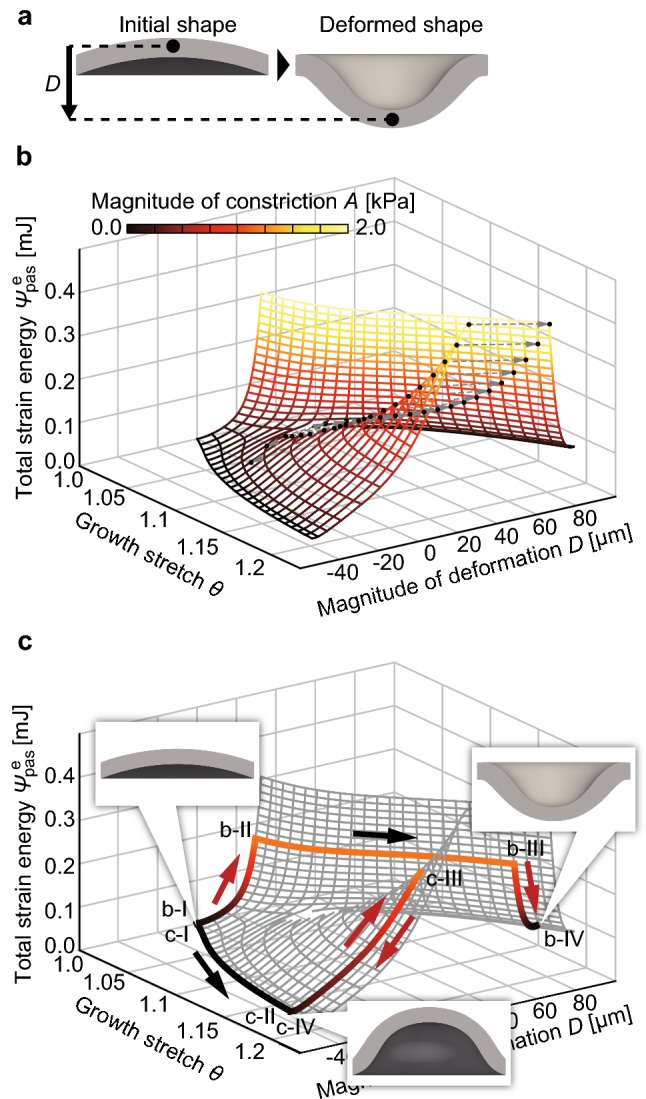
Energy landscape of morphogenesis of the sheet-like tissue. **a** Definition of deformation *D* [μm]. **b** The overview of the energy landscape, which shows the relationship between the strain energy $\varPsi_{\text{pas}}^{\text{e}}$ [mJ], deformation *D* [μm], and growth stretch *θ*. Curves drawn on the surface of the landscape indicate paths of constant growth stretch $\theta$ and magnitude of constriction *A* [kPa]. Dotted lines connect two points before and after a snap through buckling. The color map represents the magnitude of constriction *A* [kPa]. **c** The paths in the two cases where constriction occurred before and after growth. The transitions of the tissue state in these simulations are represented as the colored paths on the energy landscape. The snapshots of the tissue shown in the energy landscape represent the tissue morphologies at each point

The morphological change of the sheet-like tissue when the tissue constricted before growth is shown in Fig. 2b. When the tissue constricted from the initial state, its central region became concave (Fig. 2b-II). Throughout tissue growth, the tissue continued to deform to form a concave shape (Fig. 2b-III). Even after the constriction was released, the tissue kept its concave shape (Fig. 2b-IV). The result for when tissue was constricted after growth is shown in Fig. 2c. Tissue growth induced a convex shape (Fig. 2c-II). Next, when the tissue constricted, the central region of the tissue became concave (Fig. 2c-III). However, when constriction was released, the tissue regained its convex shape (Fig. 2c-IV). These results show that the qualitatively different morphologies depend on the order of growth and constriction.

#### Energy landscape demonstrating the overall view of morphogenesis

To understand the mechanism of how the history of growth and constriction results in the qualitatively different morphologies, we captured the overall view of the morphogenetic process. Here, we plotted an energy landscape (Fig. 2) that involves general cases in which growth and constriction occurred simultaneously; that is, any path in the growth $\theta$ and the constriction *A* space. The total strain energy $\varPsi_{\text{pas}}^{\text{e}}$ [mJ] stored in the hyperelastic tissue was quantified. Deformation *D* [μm] was defined as the displacement of the center of the sheet-like tissue from the initial shape (Fig. 2a).

The energy landscape is shown in Fig. 2b. Curves drawn on the surface of the landscape indicate the paths of the state of tissues with constant growth stretch $\theta$ and the magnitude of constriction *A*. The color map represents the magnitude of constriction *A.* The landscape was branched, and its surface became discontinuous as growth proceeded; therefore, the tissue after growth had two local stable states.

From this energy landscape, we can understand the mechanism of history-dependent morphogenesis observed in the previous two cases by drawing the corresponding paths (Fig. 2c). The Roman numbers in Fig. 2c indicate the same condition shown in Fig. 1b, c. The tissue state in these two cases followed different paths in the energy landscape. When the constriction was released, each state of the tissue attained a different local stable state, indicating the mechanism behind the qualitatively different morphologies formed depending on the history of growth and constriction.

**Fig. 3:**
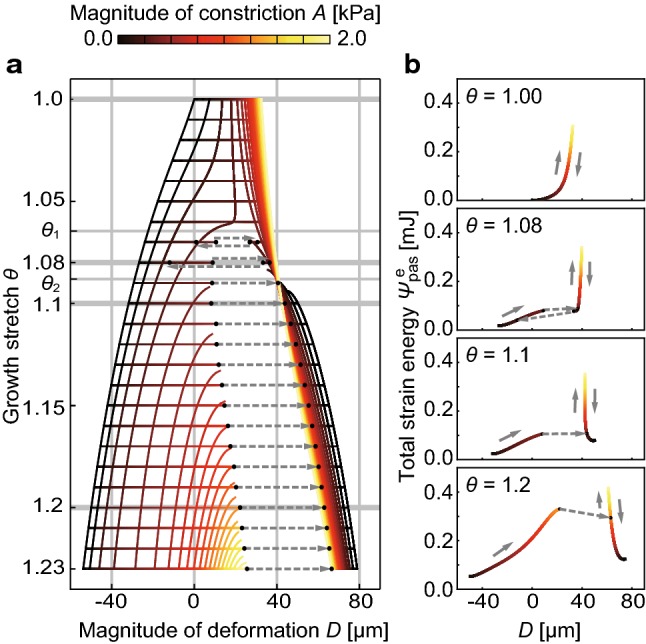
Projections of the energy landscape to the *D*–*θ* plane and *D*–$\varPsi_{\text{pas}}^{\text{e}}$ plane. **a** The relationship between the deformation *D* and growth stretch $\theta$. $\theta_{1}$ and $\theta_{2}$ are the values at which tissue deformations qualitatively changed. The color map represents the magnitude of constriction *A.***b** Typical changes in the strain energy $\varPsi_{\text{pas}}^{\text{e}}$ and deformation *D* while the growth stretch takes a constant value $\theta = 1.0, 1.08, 1.1, \;{\text{and}}\;1.2$. The arrows indicate the transition of the tissue state when the magnitude of constriction *A* increases and then releases

The projection of the energy landscape to the *D*–*θ* plane is displayed in Fig. 3a. Depending on the value of the growth stretch $\theta$ ($\ge \,1$), the tissue exhibited three qualitatively different types of behavior. The first type was when the growth stretch $\theta$ was in the region $1.0 \le \theta < \theta_{1}$; there was one stable state, and the tissue state continuously changed with changes in growth and constriction. The second type was when the growth stretch $\theta$ was in the region $\theta_{2} < \theta$; there were two stable states, and these values underwent discontinuous changes. In the process of increase in constriction, a snap through buckling, which is a sudden increase in deformation with a small increase in constriction, occurred. When the growth stretch $\theta$ was in the region $\theta_{1} \le \theta \le \theta_{2}$, which is the intermediate region of the above two types, there was only one stable state at the constriction-released state, although buckling occurred when constriction increased and released—this represents the third type.

The typical changes in strain energy and deformation with constant growth stretches $\theta = 1.0, 1.08, 1.1, \;{\text{and}}\;1.2$ are shown in Fig. 3b. The arrows in these figures represent the transition when constriction is increased and then released. In the case of growth stretch $\theta = 1.1\;{\text{and}}\;1.2$, snap through buckling occurred when the strain energy reached a certain magnitude. This energy barrier in the later stage ($\theta = 1.2$) is larger than that in the earlier stage ($\theta = 1.1$), suggesting that the tissue morphologies become harder to change to the qualitatively different morphologies. In addition, after buckling, transition with buckling no longer occurred. This indicated the irreversibility of morphogenesis.

The energy landscape demonstrated the mechanical background of emergence of qualitatively different morphologies. The energy landscape branched in the growth process and involved two local stable states, indicating that the tissue has the potential to deform to qualitatively different morphologies. In addition, paths on the surface of the landscape represented the transition of the tissue state depending on the history of growth and constriction.

### Application to luminal folding in the initial stage of intestinal villus formation

#### Simulation conditions

As a realistic biological application, we applied the energy landscape approach to the morphogenesis of intestinal villus in vertebrates. In the initial stage of villus formation, the intestinal tube folds into longitudinal ridges with differential growth of the inner epithelial and mesenchymal layers, which is constrained by the outer circumferentially oriented muscle layer (Shyer et al. 2013; Huycke and Tabin 2018).

**Fig. 4:**
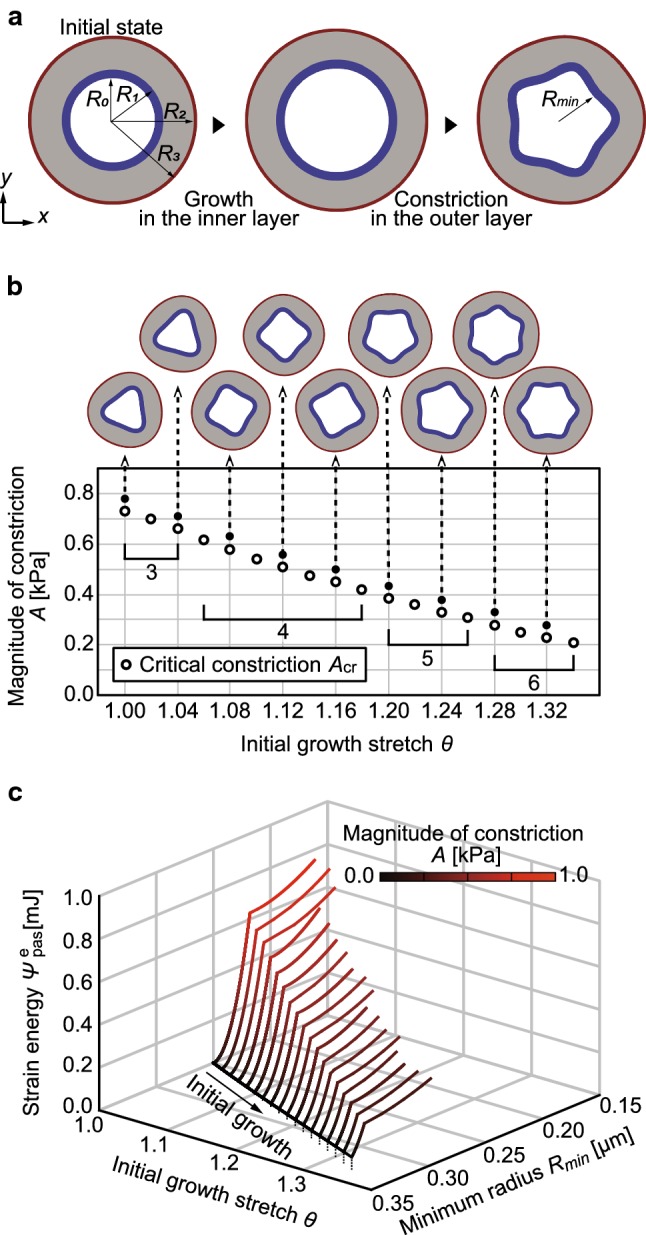
Application to luminal folding in the initial stage of intestinal villus formation. **a** Typical luminal folding process induced by circumferential growth in the inner layer followed by circumferential constriction in the outer layer. The inner layer in blue is the epithelium. The middle layer in gray is the mesenchyme. The outer layer in red is the muscle. **b** Critical magnitude of constriction $A_{\text{cr}}$, at which the tube folded, as shown in open circles. Snapshots pointed by arrows are the morphologies at each solid circle. **c** Energy landscape of luminal folding, which shows the relationship between the total strain energy in the inner layer $\varPsi_{\text{pas}}^{\text{e}}$ [mJ], minimum radius of the inner layer $R_{ \hbox{min} }$ [μm], and growth stretch $\theta$. The color map represents the magnitude of constriction *A* [kPa]

**Fig. 5:**
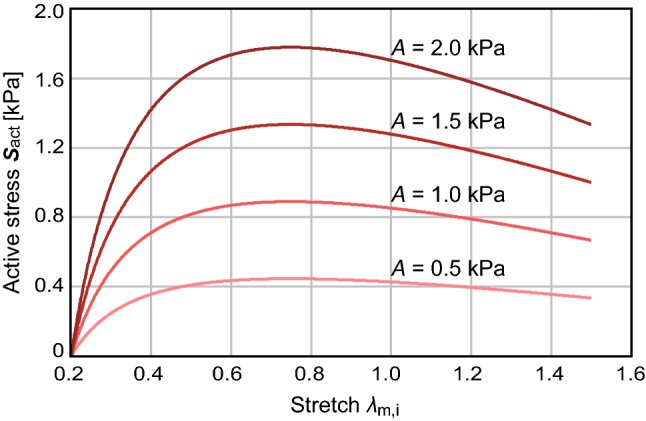
Constitutive relationship between active second Piola–Kirchhoff stress ${\mathbf{S}}_{\text{act}}$ and stretch $\lambda_{{{\text{m}},i}}$. The upper and lower limits of stretch in Eq. (9) are $\lambda_{ \hbox{max} } = 1.5$ and $\lambda_{0} = 0.2$, respectively

We performed FEAs for luminal folding of intestinal tube following previously reported analyses (Shyer et al. 2013; Goriely 2017). The FE model of the intestinal tube consists of an inner layer (epithelium), a middle layer (mesenchyme), and an outer layer (muscle) in Fig. 4a. In reference to the experimental and mathematical analyses of villus formation in chick gut (Shyer et al. 2013), we assumed that the inner layer is 12 times stiffer than the middle layer and that the outer layer has the same stiffness as the middle layer. The elastic parameters in Eq. (5) are $\lambda_{\text{in}} = 1.36$ kPa and $\mu_{\text{in}} = 0.583$ kPa in the inner layer, and $\lambda_{\text{mid}} = \lambda_{\text{out}} = \lambda_{\text{in}} /12$ and $\mu_{\text{mid}} = \mu_{\text{out}} = \mu_{\text{in}} /12$ in the middle and outer layers, respectively. We set radius of boundaries between each layer *R*_0_ = 0.25 μm, *R*_1_ = 0.30 μm, *R*_2_ = 0.48 μm, and *R*_3_ = 0.50 μm in the initial configuration (Fig. 4a). For simplicity, we applied the plane strain condition in FEAs.

We explored luminal folding caused by growth in the inner layer and constriction of the outer layer. To test the effect of differential growth on folding, we assumed no growth in the middle and outer layers and circumferential growth in the inner layer. The growth direction $\varvec{\zeta}$ in Eq. (3) was circumferential. An increase in growth stretch $\theta \left( { \ge \,1} \right)$ in Eq. (3) represents circumferential growth in the inner layer. The contractility of muscle layer was modeled as circumferential constriction in the outer layer. To represent circumferential constriction, both contractile directions $\varvec{m}_{0,1}$ and $\varvec{m}_{0,2}$ in Eq. (7) were circumferential. An increase in the magnitude of constriction $A \left( { \ge \,0\;{\text{kPa}}} \right)$ represents circumferential constriction in the outer layer. The parameters in Eq. (8) were $\lambda_{ \hbox{max} } = 1.5$ and $\lambda_{0} = 0.2$.

#### Energy landscape of luminal folding caused by growth and constriction

To illustrate the energy landscape in morphogenesis of the intestinal tube, we performed FEAs by increasing the initial growth stretch $\theta$ in the inner layer followed by the increase in magnitude of constriction $A$ in the outer layer sequentially. All the analyses of luminal folding were conducted in this order. The typical deformation process is shown in Fig. 4a. With an increase in constriction of the outer layer after the growth of the inner layer, the tube folded into ridges. Since we focused on the initial folded shapes, the deep folds and ridges observed in the model of Shyer et al. (2013) were not reproduced in this study. The critical constriction $A_{\text{cr}}$, at which the tube folded, is plotted as a function of growth stretch $\theta$ in Fig. 4b. An increase in the initial growth stretch resulted in a decrease in the critical constriction, leading to an increase in the number of ridges (snapshots in Fig. 4b). This relationship between the initial growth stretch and number of ridges was in agreement with actual intestinal morphogenesis (Shyer et al. 2013; Huycke and Tabin 2018).

To capture the overall view of luminal folding, we plotted an energy landscape by exploring the transition of strain energy $\varPsi_{\text{pas}}^{\text{e}}$ in the inner layer during its deformation (Fig. 4c). A variety of luminal folding was represented on the energy landscape. The minimum radius of the inner layer $R_{ \hbox{min} }$ in Fig. 4a was used to represent the magnitude of deformation. As the magnitude of constriction $A$ increased, the minimum radius of the inner layer $R_{ \hbox{min} }$ decreased and the strain energy $\varPsi_{\text{pas}}^{\text{e}}$ in the inner layer increased. The critical energy, which is strain energy at folding point, decreased as the initial growth stretch increased. This indicates that as the initial growth stretch increases, the tube becomes easy to fold with small energy supply, that is, luminal folding becomes robust with growth of the inner layer. By illustrating the energy landscape of luminal folding, we showed the overall view including the formation of various folded shape and robustness of these morphogenesis.

## Discussion

The fundamental characteristics of tissue morphogenesis involve the coexistence of various morphologies and the robustness of the morphogenetic process. Herein, we proposed a novel approach to capture the essential behaviors of tissue morphogenesis from a mechanical viewpoint. The approach involves visualization of the energy landscape of morphogenetic processes induced by admissible histories of cellular activities. This approach was applied to investigate the morphogenesis of a sheet-like tissue with curvature, where it deformed to a concave or convex morphology depending on the history of growth and constriction. The energy landscape enabled us to understand how the qualitatively different morphologies were formed by mechanical bifurcation and how robust to the fluctuations each formation process was.

The energy landscape of morphogenesis shows the emergence of qualitatively different morphologies and the robustness of morphogenesis to the fluctuation simultaneously. The qualitatively different morphologies are produced by bifurcation of the local stable state in the energy landscape. As the growth of the sheet-like tissue proceeds, the energy landscape becomes discontinuous, and two local stable states corresponding to the concave and convex morphologies emerge. This indicates the sheet-like tissue has a potential to form qualitatively different morphologies through growth and constriction. The depth and steepness of the valley in the energy landscape near the stable states represent the degree of robustness to fluctuations in multicellular activities. As the growth of the sheet-like tissue proceeds, the energy barrier between the two local stable states became larger, indicating these states become harder to transit each other. The proposed approach using the energy landscape during tissue morphogenesis is an analogy of an epigenetic landscape. As an attempt to understand developmental events from a macroscopic viewpoint, Waddington proposed the epigenetic landscape (Waddington 1957), where the diversity of differentiated cells through complex molecular interactions is represented as an emergence of multiple valleys. As the epigenetic landscape is the basis of the discussion for the diversity and robustness of cell differentiation in various studies, our approach can help us understand tissue morphogenesis from a mechanical viewpoint.

This approach can be useful to understand the mechanism of morphogenesis observed in experimental studies, which have observed temporal behavior of multicellular proliferation and constriction and subsequent formation of various tissue morphologies (Seher and Leptin 2000). For example, during furrow formation in early *Drosophila* embryos, in which constriction drives tissue invagination, the qualitatively different tissue morphologies arise from the timing of inhibition of cell constriction. While inhibition of cell constriction at the early formation stage resulted in the failure of invagination, the tissue invaginated even with the inhibition in the late stage (Royou et al. 2004; Krajcovic and Minden 2012). This difference in the tissue morphologies can be explained based on the energy landscape illustrated in this study. With a decrease in constriction, the tissue became concave in the early stage of growth, while it remained convex in the later stage (Fig. 3b). In addition, we showed the example of application to realistic morphogenesis. We applied the energy landscape approach to luminal folding in the initial stage of intestinal villus formation (Fig. 4c). By describing the energy landscape of the intestinal tube, we showed the overall view including the formation of various folded shapes and robustness of these morphogenesis. This approach can be applied to other tissue folding processes, such as wrinkling of the brain tissue in development (Budday et al. 2015; Goriely et al. 2015) and luminal folding of airway in asthma (Goriely 2017).

Morphogenesis usually involves spatial variations in growth and constriction, such as spatial pattern (Filas et al. 2012a, b; Kondo and Hayashi 2015; Misra et al. 2016; Inoue et al. 2017) and fluctuation at the tissue and cell levels, which possibly induce different morphogenetic processes. To investigate qualitative difference in morphogenesis, it will be useful to compare these energy landscapes for each spatial pattern. In addition, through statistical analysis, the robustness of morphogenesis against spatial fluctuation can be evaluated.

We captured the essential behavior of tissues during morphogenesis in a simplified way by targeting morphogenesis of the sheet-like tissue caused by cell proliferation and constriction. To understand actual morphogenesis, further development is required to investigate actual morphogenesis by modeling two factors influencing the shape of the energy landscape itself. The first is the plastic deformation of tissues induced by cell migration (Scarpa and Mayor 2016), such as cell rearrangement resulting in energy dissipation and stress relaxation (David et al. 2014; Etournay et al. 2015). This characteristic behavior of multicellular tissue would be modeled as a change in stress-free configuration. The second is a mechanical constraint, such as extracellular matrix surrounding a growing tissue, that determines the direction of invagination (Huang et al. 2011; Oltean et al. 2016). Further analysis is required to investigate the effects of mechanical constraints modeled as boundary conditions on tissue deformation. By improving our understanding of actual morphogenesis through the above developments, the mechanical approach proposed in this study will be helpful to understand how various morphologies are formed and how tissues reproducibly achieve specific morphologies.

## Appendix

The active second Piola–Kirchhoff stress tensor ${\mathbf{S}}_{\text{act}}$ is derived from $\psi_{\text{act}}^{\text{e}}$ as follows (Kida and Adachi 2015):

9 $${\mathbf{S}}_{\text{act}} = 2\frac{{\partial \psi_{\text{act}}^{\text{e}} }}{{\partial {\mathbf{C}}^{\text{e}} }} = \mathop \sum \limits_{i = 1}^{2} \frac{A}{{\lambda_{{{\text{m}},i}} }}\left\{ {1 - \left( {\frac{{\lambda_{ \hbox{max} } - \lambda_{{{\text{m}},i}} }}{{\lambda_{ \hbox{max} } - \lambda_{0} }}} \right)^{2} } \right\}\varvec{m}_{0} \otimes \varvec{m}_{0} .$$

The constitutive relationship between ${\mathbf{S}}_{\text{act}}$ and stretch $\lambda_{{{\text{m}},i}}$ along the constriction direction $\varvec{m}_{i}$ is plotted in Fig. 5. The upper and lower limits of stretch in Eq. (9) were set to $\lambda_{ \hbox{max} } = 1.5$ and $\lambda_{0} = 0.2$, respectively.
